# *Trichoderma harzianum* favours the access of arbuscular mycorrhizal fungi to non-host Brassicaceae roots and increases plant productivity

**DOI:** 10.1038/s41598-019-48269-z

**Published:** 2019-08-12

**Authors:** Jorge Poveda, Rosa Hermosa, Enrique Monte, Carlos Nicolás

**Affiliations:** 1 Spanish-Portuguese Institute for Agricultural Research (CIALE), Department of Botany and Plant Physiology, University of Salamanca, Salamanca, Spain; 2 Spanish-Portuguese Institute for Agricultural Research (CIALE), Department of Microbiology and Genetics, University of Salamanca, Salamanca, Spain

**Keywords:** Arbuscular mycorrhiza, Plant breeding

## Abstract

The family Brassicaceae includes plants that are non-host for arbuscular mycorrhizal fungi (AMF) such as the model plant *Arabidopsis thaliana* (arabidopsis) and the economically important crop plant *Brassica napus* (rapeseed). It is well known that *Trichoderma* species have the ability to colonize the rhizosphere of Brassicaceae plants, promoting growth and development as well as stimulating systemic defenses. The aim of the present work is to ascertain that Brassicaceae plants increase productivity when AMF and *Trichoderma* are combinedly applied, and how such an effect can be ruled. This simultaneous application of a *Trichoderma harzianum* biocontrol strain and an AMF formulation produces a significant increase in the colonization by *Trichoderma* and the presence of AMF in arabidopsis and rapeseed roots, such colonization accompanied by improved productivity in both Brassicaceae species. Expression profiling of defense-related marker genes suggests that the phytohormone salicylic acid plays a key role in the modulation of the root colonization process when both fungi are jointly applied.

## Introduction

Arbuscular mycorrhizal fungi (AMF) are able to establish symbiotic relationships with the majority of terrestrial plants, including species of great economic interest in agriculture^1^. As a result, the plant benefits from improved water and nutrient uptake, while the fungus receives a place to develop the mycorrhizal structures and the uptake of sugars produced during the photosynthetic process^2–4^. AMF are also able to improve systemic plant responses to environmental^5,6^ and biotic^7,8^ stresses. However, the loss of essential genes for symbiosis during evolution^9^ has led to the inability of several flowering plant lineages, including most plants of the Brassicaceae family, to form AMF symbiotic relationships^10,11^. *Brassica* is the most important genus of the Brassicaceae because it includes crop plants such as *Brassica oleracea* (broccoli, cabbage, cauliflower), *B*. *rapa* (turnip), *B*. *nigra* (black mustard) and *B*. *napus* (rapeseed), as well as the model plant *Arabidopsis thaliana* (arabidopsis)^12^.

*Trichoderma* is a genus of soil-borne filamentous fungi widely used as a source of biocontrol agents in agriculture owing to effective antagonisitic mechanisms such as mycoparasitism, antibiosis or competition against plant pathogens and nematodes^13,14^. Some strains are able to induce plant defenses^15^ and stimulate plant growth and development^16,17^ by establishing a molecular dialogue with the roots^18,19^. Mutualistic beneficial interactions between *Trichoderma* spp. and plants such as arabidopsis, tomato, cucumber, pea or canola^20–22^ and even woody plants^23^ have been observed.

Although *Trichoderma* and AMF are commonly applied as beneficial microorganisms in agriculture, little is known about the molecular changes occurred in the plants when these fungi are used together. It has been reported that the combined application of *Trichoderma* and AMF has positive effects in the nutritional composition of several plant species including marigold, tomato, cucumber or melon^24–27^. Nevertheless, negative effects for plants have been also described when these two fungi were applied together^28,29^.

A complex regulatory network mediated by phytohormones, involving the salycilic acid (SA), jasmonic acid (JA) and ethylene (ET) pathways, plays a key role during the AMF or *Trichoderma* root colonization process^30–33^. During the first hours of plant-microbe interaction a SA-dependent response known as systemic acquired resistance (SAR) takes place in plant roots^32^. In addition, beneficial microorganisms activate the mechanism known as induced acquired resistance (ISR), mediated by JA and ET^34^. These signaling pathways can interact antagonistically or synergistically and are effective at fighting against a broad range of attackers and colonizers^35^, including the symbiotic interactions with beneficial microbes^34^. In order to reach an effective association with the plant, AMF and *Trichoderma* establish a molecular dialogue with their host that bypasses plant defenses allowing the root system to be colonized during the first hours of interaction^36,37^. This process requires microorganisms to produce and secrete small-sized molecules recognizable by plant cell receptors^19,38^. As far as *Trichoderma* is concerned, response mediated by SA is expected at the root level during the first hours of colonization^14,37^, whereas in the case of AMF, a response mediated by JA takes place^39^.

The objectives of this study have been to analyze: i) the effect of the combined application of *T*. *harzianum* T34 and an AMF formulation on fungal root colonization of two non-host (arabidopsis and rapeseed) and one host (*Solanum lycopersicum*, tomato) AMF plants, and on arabidopsis and rapeseed silique production; ii) the plant transcriptomic changes derived from the combined use of these two fungal inocula; and iii) the role of SA and JA on fungal root colonization when T34 and AMF are applied together.

## Results

### Arabidopsis and rapeseed production

**Figure 1:**
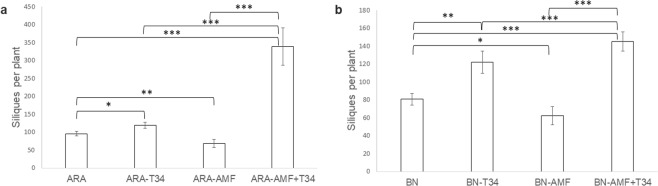
Number of siliques produced by arabidopsis (**a**) and rapeseed (**b**) plants. Arabidopsis (ARA) and rapeseed (BN) inoculated with *T*. *harzianum* T34 (-T34) and AMF (-AMF). Each value represents the average of 45 plants from 3 independent experiments (15 plants each), with their standard deviation. Two-way analysis of variance (ANOVA) was performed, followed by Sidak’s multiple comparison test, indicating significant differences as follows: *P < 0.05; **P < 0.01; ***P < 0.001.

The number of siliques was used to determine the productive capacity of the different Brassicaceae plants used. The production of siliques was significantly greater in arabidopsis and rapeseed plants inoculated with *T*. *harzianum* T34 in contrast to the untreated control plants (Fig. 1), whereas production was lower in plants only challenged with AMF. However, the highest number of siliques was recorded for arabidopsis and rapeseed plants treated with AMF plus T34.

### Root colonization

Using a quantitative real-time PCR (qPCR) analysis, the colonization of *T*. *harzianum* T34 and AMF when applied together or separately was evaluated in arabidopsis, rapeseed and tomato roots. The T34 and/or AMF colonization rates are shown in Table 1. For single inoculations, DNA of T34 was detected in arabidopsis, rapeseed and tomato roots, whereas DNA of AMF was only detected in tomato roots. The combined application of AMF and T34 led to significantly increased levels of *T*. *harzianum* in both arabidopsis and rapeseed roots and an opposite effect in tomato roots. After the combined inoculation of T34 and AMF significantly increased levels of AMF were measured in tomato roots, as well as the presence of AMF was detected in roots of the two Brassicaceae plants.

**Table 1:** Arabidopsis (ARA), rapeseed (BN) and tomato (TOM) root fungal colonization by *T*. *harzianum* T34 (+T34) and AMF (+AMF).

Treatments	Quantified fungi	Plant			Fungi			Ratio^c^
		Ct	SD	Qty^a^	Ct	SD	Qty^b^	
ARA + T34	T34	19.65	0.08	2.46	25.68	0.15	1.11	0.45 ± 0.02
ARA + AMF	AMF	19.89	0.06	2.17	—	—	—	—
ARA + T34 + AMF	T34	19.78	0.10	2.28	24.55	0.29	2.03	0.89 ± 0.06*
	AMF				28.82	0.23	2.74	0.12 ± 0.01
BN + T34	T34	21.32	0.12	0.79	27.95	0.25	0.30	0.38 ± 0.01
BN + AMF	AMF	21.28	0.05	0.84	—	—	—	—
BN + T34 + AMF	T34	20.98	0.03	0.90	26.47	0.21	0.62	0.69 ± 0.05*
	AMF				30.14	0.19	0.08	0.09 ± 0.01
TOM + T34	T34	20.56	0.06	1.25	26.38	0.22	0.67	0.54 ± 0.04
TOM + AMF	AMF	20.69	0.11	1.20	27.53	0.12	0.78	0.65 ± 0.04
TOM + T34 + AMF	T34	20.75	0.02	1.19	28.79	0.20	0.16	0.14 ± 0.02*
	AMF				26.83	0.17	0.99	0.83 ± 0.07*

^a^Quantity of plant DNA (ng) referred to *actin* gene.

^b^Quantity of fungi DNA (ng) referred to *Trichoderma actin* gene and AMF 18 s rRNA.

^c^Proportion of fungal DNA vs. plant DNA. Values are the means of three root pools (five plants each one) from three independent experiments with the corresponding standard deviations, and values followed by * are significantly different (P < 0.05) regarding the single application of T34 or AMF.

— Absence of amplification.

Quantification of fungal DNA in arabidopsis (3-week-old plants) roots was performed by qPCR, using the *actin* genes of *Trichoderma* and arabidopsis, and the 18S rRNA for AMF.

To determine the AMF species in these plants, DNA extracted from root samples was amplified and sequenced using genus-specific primers, and a sequence analysis showed that *Rhizophagus fasciculatus* and *Rhizophagus irregularis* were the only fungal species present in roots of arabidopsis and rapeseed plants, respectively (data not shown).

### Effect of T34 and AMF combined application in local defense of arabidopsis and tomato plants

**Figure 2:**
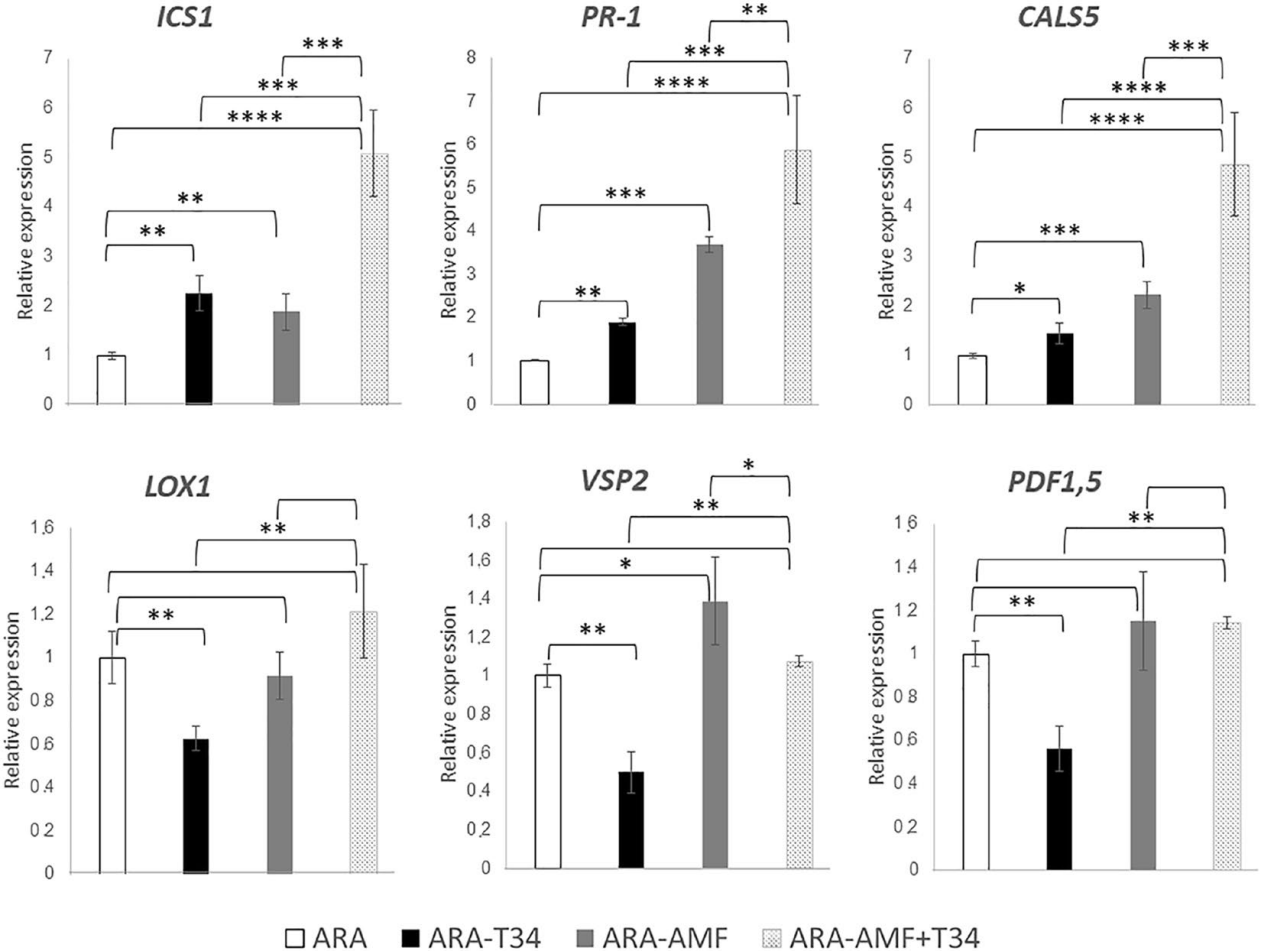
Real time reverse transcription polymerase chain reaction (qRT-PCR) analysis of some defense genes in roots of 3 week-old *A*. *thaliana* plants inoculated with *T*. *harzianum* T34 and/or AMF. Genes of the isochorismate synthase 1 (*ICS1*), pathogenesis-related protein 1 (*PR-1*), callose synthase 5 (*CALS5*), lipoxygenase 1 (*LOX1*), vegetative storage protein (*VSP2*) and plant defensin 1.5 (*PDF1*.*5*). Values correspond to relative measurements against arabidopsis grown in the absence of the fungus (2^−ΔΔCt^ = 1). The arabidopsis *actin* gene was used as an internal reference. Bars represent standard deviations of the means of three root pools of five plants each one collected from three independent experiments. Two-way analysis of variance (ANOVA) was performed, followed by Sidak’s multiple comparison test, indicating significant differences as follows: *P < 0.05; **P < 0.01; ***P < 0.001; ****P < 0.0001.

**Figure 3:**
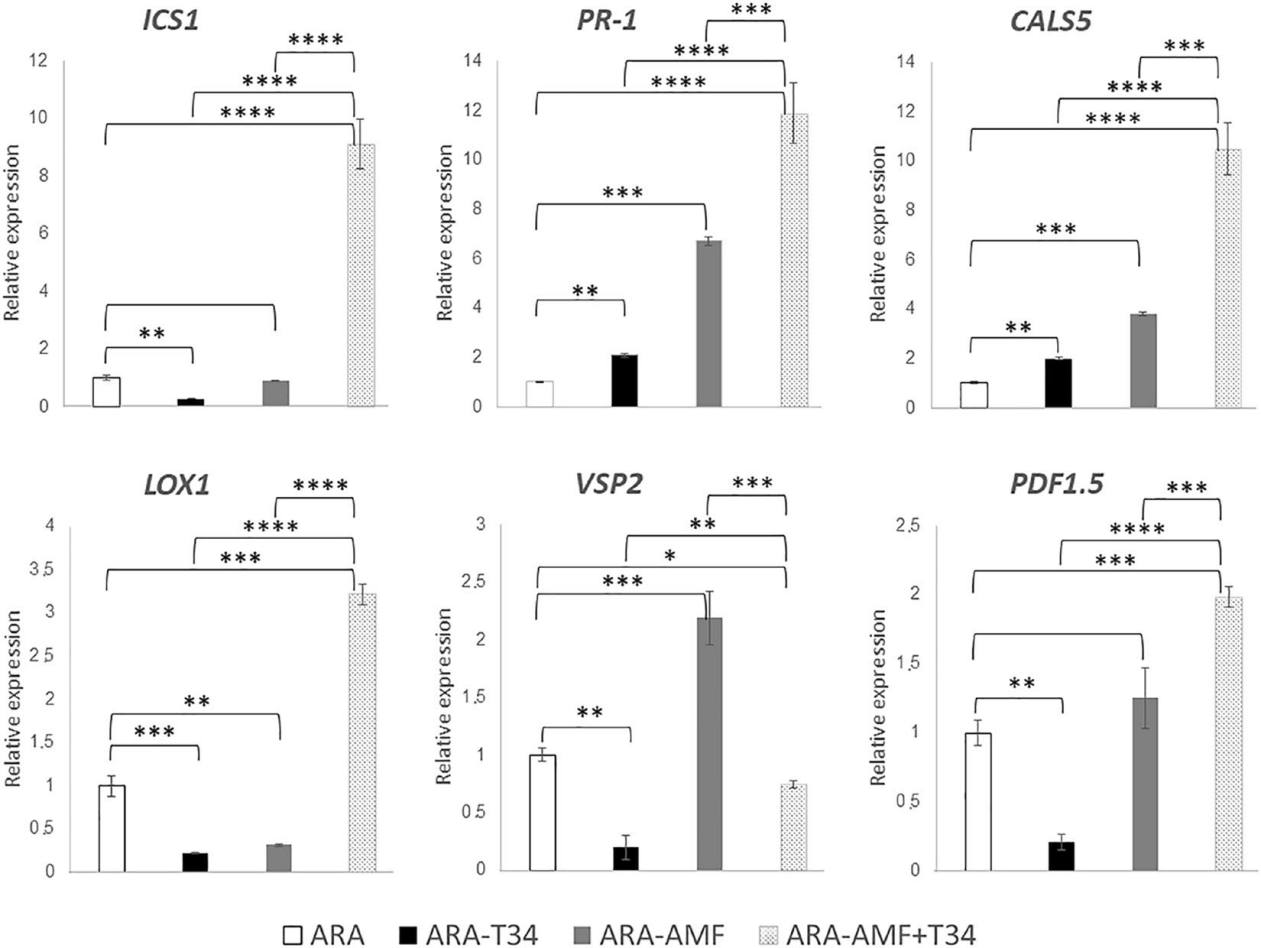
Real time reverse transcription polymerase chain reaction (qRT-PCR) analysis of some defense genes in roots of 5 week-old *A*. *thaliana* plants inoculated with *T*. *harzianum* T34 and/or AMF. Genes of the isochorismate synthase 1 (*ICS1*), pathogenesis-related protein 1 (*PR-1*), callose synthase 5 (*CALS5*), lipoxygenase 1 (*LOX1*), vegetative storage protein (*VSP2*) and plant defensin 1.5 (*PDF1*.*5*). Values correspond to relative measurements against arabidopsis grown in the absence of the fungus (2^−ΔΔCt^ = 1). The arabidopsis *actin* gene was used as an internal reference. Bars represent standard deviations of the means of three root pools of five plants each one collected from three independent experiments. Two-way analysis of variance (ANOVA) was performed, followed by Sidak’s multiple comparison test, indicating significant differences as follows: *P < 0.05; **P < 0.01; ***P < 0.001; ****P < 0.0001.

**Figure 4:**
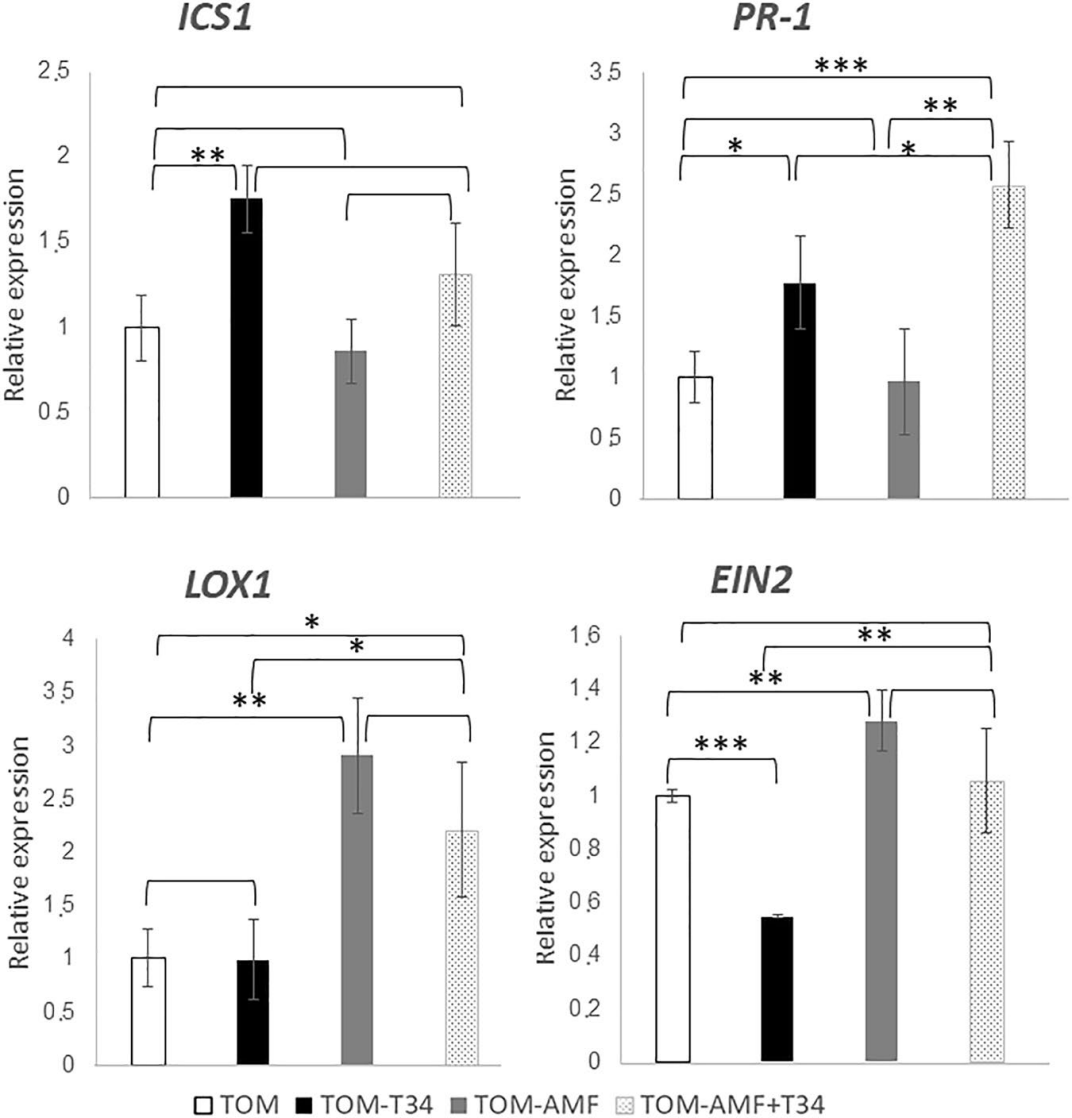
Real time reverse transcription polymerase chain reaction (qRT-PCR) analysis of expression of some defense genes in roots of 4 weeks-old tomato plants inoculated with *T*. *harzianum* T34 and/or AMF. Genes of the isochorismate synthase 1 (*ICS1*), pathogenesis-related protein 1 (*PR-1*), lipoxygenase 1 (*LOX1*) and ethylene signaling protein (*EIN2*). Values correspond to relative measurements against tomato grown in the absence of the fungus (2^−ΔΔCt^ = 1). The tomato *actin* gene was used as endogenous reference control. Bars represent standard deviations of the means of three root pools of five plants each one collected from three independent experiments. Two-way analysis of variance (ANOVA) was performed, followed by Sidak’s multiple comparison test, indicating significant differences as follows: *P < 0.05; **P < 0.01; ***P < 0.001; ****P < 0.0001.

**Figure 5:**
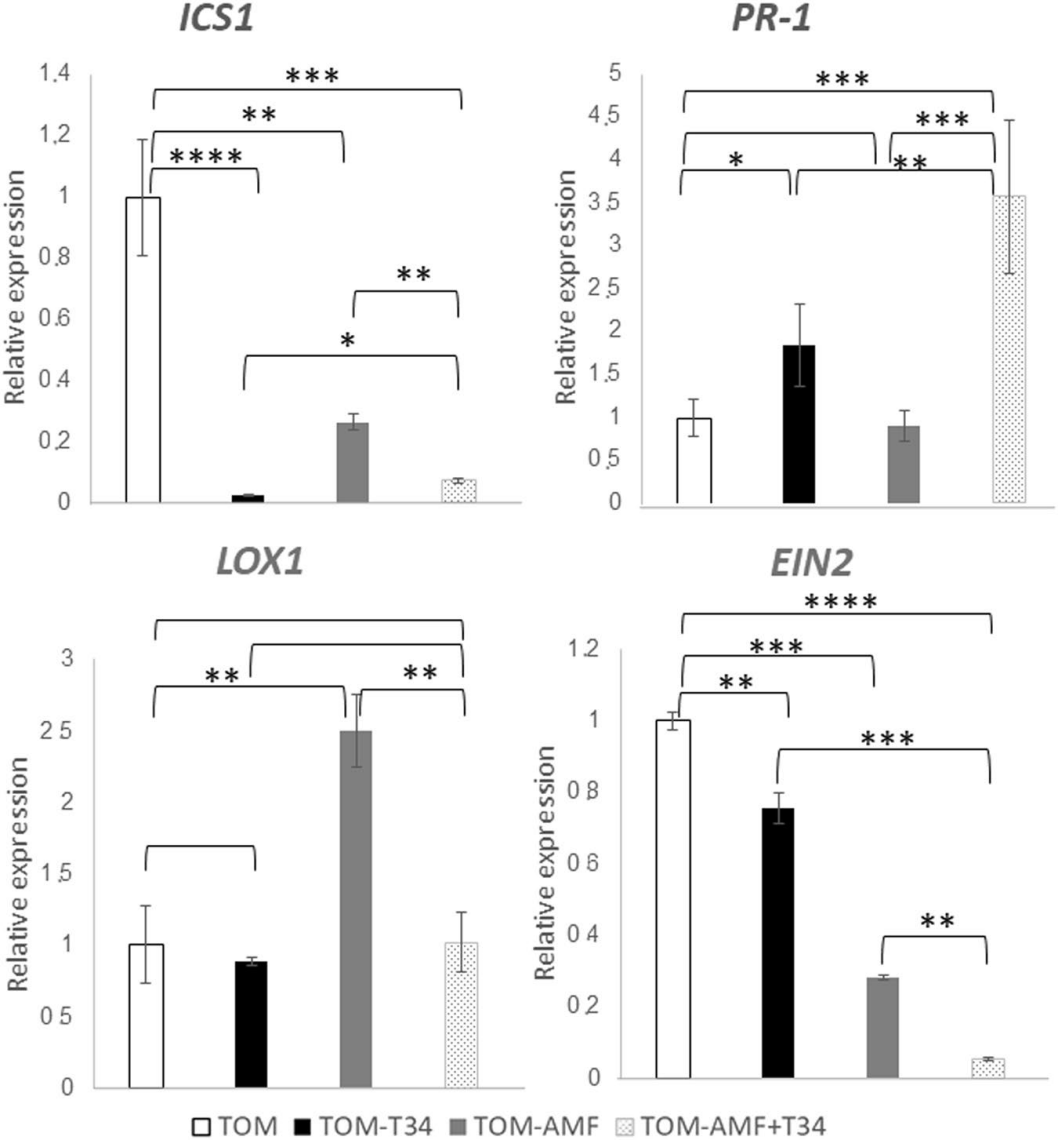
Real time reverse transcription polymerase chain reaction (qRT-PCR) analysis of expression of some defense genes in roots of 7 weeks-old tomato plants inoculated with *T*. *harzianum* T34 and/or AMF. Genes of the isochorismate synthase 1 (*ICS1*), pathogenesis-related protein 1 (*PR-1*), lipoxygenase 1 (*LOX1*) and ethylene signaling protein (*EIN2*). Values correspond to relative measurements against tomato grown in the absence of the fungus (2^−ΔΔCt^ = 1). The tomato *actin* gene was used as endogenous reference control. Bars represent standard deviations of the means of three root pools of five plants each one collected from three independent experiments. Two-way analysis of variance (ANOVA) was performed, followed by Sidak’s multiple comparison test, indicating significant differences as follows: *P < 0.05; **P < 0.01; ***P < 0.001; ****P < 0.0001.

To explore whether the response of host and non-host mycorrhizal plants to combined or separated applications of AMF and T34 involved different local defense responses, we analyzed by qPCR the expression levels of defense-related phytohormone marker genes genes at two time points, in roots untreated or previously inoculated with T34, AMF and AMF plus T34. In the case of Arabidopsis, roots of 3-week-old plants (Fig. 2), just one week after fungal inoculation, and roots of 5-week-old-plants (Fig. 3) were analyzed; in the case of tomato, roots of 4-week-old plants (Fig. 4), just one week after fungal inoculation, and roots of 7-week-old-plants (Fig. 5) were analyzed. Compared to the arabidopsis control plants, the application of T34 alone increased the expression levels of *PR-1* and *CALS5*, both SA-responsive markers, and *ICS1*, involved in SA biosynthesis just one week after fungal inoculation (Figs 2 and 3). By contrast, the expression levels of *VSP2* and *PDF1*.*5* genes, both JA-responsive markers, were reduced at both time points, whereas expression of *LOX1*, involved in the JA biosynthetic pathway, were reduced only in 5-week-old-plants (Figs 2 and 3). Although the inoculation of AMF alone also increased the expression levels of the two marker genes of the SA-responsive pathway at both time points, and *ICS1*, one week after T34 application, (Figs 2 and 3) it was accompanied by increased expression levels of *VSP2* and a reduction of *LOX1* levels in roots of 7-week-old-plants (Figs 2 and 3). The combined application of AMF and T34 caused significant changes in the expression of the six genes analyzed, increasing the transcript levels of all of them, except for those of *VSP2*, and *PDF1*.*5* one week after fungal application (Figs 2 and 3). These profiles are significant not only in comparison with the control condition, but also with those from arabidopsis plants treated with single applications of T34 or AMF. Changes in SA- and JA-signaling defense pathways in arabidopsis roots should affect fungal colonization rates when AMF and T34 are applied together.

Compared with tomato control plants (Figs 4 and 5), the single application of *T*. *harzianum* increased the expression levels of *ICS1* and *PR-1*, one week after T34 application and reduced those of *ICS1* and *EIN2*, a major regulator of the ET signaling pathway, in roots of 7-week-old-plants, while the expression of *LOX1* did not significantly change. The application of AMF alone caused increased expression of *LOX1* but did not modify the *PR-1* expression at both time points (Figs 4 and 5). Compared to the control, the combined application of AMF plus T34 showed a similar gene expression profile to that observed in tomato plants treated with T34 which would be indicating the prevalence of *Trichoderma* over the AMF effect.

### Colonization of SA- and JA-impaired arabidopsis mutants

In order to analyze the role played by the SA- and JA-signaling defense pathways in the process of fungal colonization of a non-host AMF plant such as arabidopsis we employed mutants impaired in SA biosynthesis (*sid2*) and in JA response (*coi1-30*).

The application of AMF plus T34 caused the death of *sid2* plants during the floral development stage (data not shown). Roots were collected 10 days after T34 application in order to perform a comparative analysis of the colonization pattern by *T*. *harzianum* in the wild type and the two mutant lines of arabidopsis. When T34 was applied alone, a significant increase of fungal DNA was detected in *sid2* mutant roots as compared with that of the Col-0 wild type. The highest levels of fungal DNA was detected in the roots of plants treated with the AMF plus T34 condition (Table 2). With respect to the *coi1-30* mutant, the colonization pattern of T34 was similar to that observed in Col-0 roots. As expected for a non-host AMF plant, no fungal DNA was detected in Col-0 roots inoculated with AMF alone, although AMF DNA was detected in the *sid2* mutant inoculated only with AMF. In addition, the highest levels of AMF DNA were detected in *sid2* plants inoculated with AMF plus T34. The fungal root colonization ability was the same for both the mutant *coi1-30* and the Col-0 wild type.

**Table 2:** Analysis of fungal root colonization by *T*. *harzianum* T34 (+T34) and AMF (+AMF) in Arabidopsis wild-type Col-0 (COL) and defensive mutants *sid2* (SID) and *coi1-30* (COI).

Treatments	Quantified fungi	Plant			Fungi			Ratio^c^
		Ct	SD	Qty^a^	Ct	SD	Qty^b^	
COL + T34	T34	20.32	0.05	1.98	26.37	0.12	0.71	0.36 ± 0.03
COL + AMF	AMF	19.99	0.07	2.04	—	—	—	—
COI + T34	T34	19.96	0.09	2.08	26.45	0.17	0.69	0.33 ± 0.01
COI + AMF	AMF	19.87	0.10	2.19	—	—	—	—
COI + T34 + AMF	T34	20.12	0.08	2.00	25.59	0.14	1.37	0.68 ± 0.04*
	AMF				29.97	0.09	0.20	0.10 ± 0.02
SID + T34	T34	20.08	0.05	2.01	25.03	0.12	1.75	0.87 ± 0.05*
SID + AMF	AMF	19.89	0.07	2.18	27.61	0.18	0.76	0.35 ± 0.06
SID + T34 + AMF	T34	20.02	0.03	2.03	24.68	0.11	1.97	0.96 ± 0.03*
	AMF				26.59	0.12	1.13	0.56 ± 0.07*

^a^Quantity of plant DNA (ng) referred to arabidopsis *actin* gene.

^b^Quantity of fungi DNA (ng) referred to *Trichoderma actin* gene and AMF 18s rRNA.

^c^Proportion of fungal DNA vs. plant DNA. Values are the means of three root pools (five plants each one) from three different experiments with the corresponding standard deviations, and values followed by * are significantly different (P < 0.05) regarding the single application of T34 or AMF.

— Absence of amplification.

Quantification of fungal DNA in arabidopsis (5-week-old plants), rapeseed (10-week-old plants) and tomato (7-week-old-plants) roots was performed by qPCR, using the *actin* genes of *Trichoderma*, arabidopsis, rapeseed and tomato, and the 18S rRNA for AMF.

## Discussion

It is well documented that the application of beneficial fungi, such as AMF and *Trichoderma* spp., improves crop productivity and reduces the use of agrochemicals and their negative effects on human health and the environment^40–42^. In the present study we found that *T*. *harzianum* T34 increases silique production in Brassicaceae plants of arabidopsis and rapeseed (Fig. 1), while AMF inoculation significantly reduces silique production. Such positive effects are in agreement with those reported for this *T*. *harzianum* strain^32^ and other strains of *T*. *atroviride* and *T*. *virens*^21^ in arabidopsis and of *T*. *asperellum* in rapeseed^42^. Negative responses to AMF application were also observed in non-host AMF plants^43,44^, including Brassicaceae^45,46^. The increased productivity in response to the combined application of AMF and *Trichoderma* in the two non-host mycorrhizal plants used in this study (Fig. 1) is in agreement with previous findings reported in marigold^24^, melon^27^, cucumber^26^ and black lentil^47^; although such positive effects are also dependent on the fungal strains used^29^. It has also been described that the combined application of AMF and organic matter may help to facilitate plant nutrient uptake^48^, although this effect has been associated to edaphic characteristics and the presence of other soil microorganisms^49^.

The higher number of siliques recorded for arabidopsis and rapeseed after the combined inoculation of AMF plus T34 could be related to the changes detected in the ability of T34 to colonize the roots of both plants, which was enhanced by the presence of AMF We were not able to observe the formation of vesicles nor arbuscules after the joint application of *T*. *harzianum* and AMFs although, interestingly, AMF DNA was detected in the roots of the two non-host mycorrhizal plants only when AMF and T34 were applied together, indicating that *T*. *harzianum* facilitates the presence of AMF in arabidopsis and rapeseed roots (Table 1). Even though some authors have reported the development of rudimentary AM phenotypes in non-mycorhizal plants^11^, without arbuscule formation^10^, it is clear that arabidopsis is not a true AMF host^11^. The beneficial effects on a host plant is not always accompanied by the formation of arbuscules or vesicles^50^ and this fact may explain the increased productivity observed in arabidopsis and rapeseed plants from the treatment T34 plus AMF (Fig. 1).

Recently it has been suggested that plants belonging to Brassicaceae have lost the ability to form AMF symbiotic relationships during evolution, probably due to the loss of AMF symbiosis-related genes^9^ and/or the ability of plants to recognize AMF effectors that play a crucial role in establishing the symbiotic association^51^. *Trichoderma* is also able to produce several secreted proteins capable of being recognized by plant receptors, which allow *Trichoderma*-plant associations to take place^19^. It has been described that during the establishment of a symbiotic association there is a transient suppression of plant defenses that facilitates *Trichoderma* penetration^19,32,37^ and that may allow the access of AMF to the Brassicaceae roots when both fungi are applied together. Moreover, it has been described that a combination of *Trichoderma* and AMF favours the germination of *Trichoderma* conidia^52^ that subsequently can parasitize the AMF^53,54^. The increased levels of T34 DNA detected in arabidopsis and rapeseed roots after T34 plus AMF application are in agreement with these two reports.

In contrast to our observations in Brassicaceae plants, the combined application of AMF and T34 in tomato was associated with increased and reduced DNA levels of AMF and T34, respectively (Table 1). These results are in line with increased AMF colonization of melon plants after the combined inoculation of *T*. *harzianum* with *Septoglomus constrictum* (formerly *G*. *constrictum*), *G*. *claroideum* or *R*. *irregularis*^27^. However, a *T*. *harzianum* root colonization decrease was also reported by these authors when this fungus was inoculated together with *F*. *mosseae*, *G*. *claroideum* or *S*. *constrictum*. The decreased levels of *T*. *harzianum* detected in tomato roots in response to the AMF plus T34 application could be due to fungal competition for space or nutrients, as previously reported between AMF and *Trichoderma*^24,28,55^, or to the AMF-induced plant defense against *Trichoderma*^56^.

Plant interactions with beneficial or pathogenic fungi triggers a complex phytohormone networking that leads to local and systemic defense responses, in which the SA- and JA/ET-dependent pathways are mainly involved^34,35^. As biotrophic fungi, *Trichoderma* and AMF are sensitive to SA-dependent defenses which prevent the fungus from entering into the vascular system^32,57^. During the early stages of the interaction, the establishment of a symbiotic relation requires the suppression of SA-dependent defense responses in the plant, which are compensated by an increase of JA-/ET-regulated defenses^7,37^. The alternation of SA- and JA-dependent defense responses leads to an undulating defensive response in the plant once the fungal colonizer has achieved an effective root colonization^57^. In the present work, we have chosen two time points. The first one week after T34 application and the second at flowering, when AMF establishment is more efficient^58^. Increased levels of SA-response genes, such as *PR-1* and *CALS5*, accompanied by decreased expression levels of the JA/ET-marker, were detected in both cases (Figs 2–5). These results indicate that a local SA response is needed to avoid massive fungal root colonization. Similar increases in *PR-1* expression have been reported in arabidopsis and tomato plants grown under *in vivo* conditions at longer time points after *Trichoderma* was applied^14,37^.

Different defense responses were observed in non-host and host mycorrhizal plants challenged with AMF (Figs 2–5). In the non-host plant, arabidopsis, a local SA- and JA-mediated response was observed. This result is consistent with the defense responses observed in incompatible associations between AMF and non-host plants and in the pea *myc*- mutant plant, which is unable to form AMF symbiosis^59^. The increased expression of *LOX1* observed in tomato roots colonized by AMF agrees with the enhancement of JA biosynthesis previously described in mycorrhized tomato roots^7,39^. In any case, it is difficult to propose a model for hormonal crosstalk in plants occurring under multi-fungal interactions^60^. We have observed in arabidopsis roots that marker genes of the JA and SA pathways overlap in the combined application of AMF and T34.

The differences observed in the colonization pattern among host and non-host AMF plants seems to be related to the expression of SA-related genes, as assessed by the use of the arabidopsis mutants impaired in SA biosynthesis (*sid2*) and JA response (*coi1-30*), respectively (Table 2). The fungal DNA levels detected in the insensitive JA mutant agreed with those observed for the wild type, indicating that the lack of responses mediated by JA does not seem necessary for the mycorrhization process. However, in the case of the SA-deficient mutant, inoculation with AMF alone leads to root colonization even in the absence of T34, suggesting that this hormone plays a crucial role in fungal colonization, at least in arabidopsis.

In conclusion: i) SA seems to be the key phytohormone to prevent mycorrhization in Brassicaceae plants; ii) the simultaneous application of *Trichoderma* and AMF produces a significant increase of the *Trichoderma* root colonization levels and favours the presence of the AMF in Brasicaceae roots; and iii) the combined application of AMF and *T*. *harzianum* leads to a significant increase in the number of siliques in Brassicaceae plants. This finding may have a significant impact from an agronomic point of view, since the combined application of both types of fungi can produce a significant increase in the productivity of this important family of plants.

## Methods

### Plant material

The *Arabidopsis thaliana* ecotype Col-0, its SA-impaired mutant *sid2* and its JA-impaired mutant *coi1-30*, as well as *Solanum lycopersicum* cv. Marmande and *Brassica napus* cv. Jura were the plants used in this study. The SA-impaired mutant *sid2* SALK_088254 was kindly provided by Dra. P. García-Agustín (University Jaume I, Castellón, Spain), and the JA-impared mutant *coi1-30* was kindly provided by Dr. Roberto Solano (CNB-CSIC, Madrid, Spain). Seeds from arabidopsis, tomato and rapeseed plants were surface-sterilized as previously described^61^.

### Fungal cultures

*Trichoderma harzianum* CECT 2413 (Spanish Type Culture Collection, Valencia, Spain) (referred to as strain T34), was used throughout this study. Strain T34 was routinely grown on potato-dextrose-agar (PDA, Sigma-Aldrich, Madrid, Spain) in the dark at 28 °C and the spores were stored at −80 °C in a 20% glycerol solution. Spores were harvested from 7-day-old PDA plates as previously described^57^, and a final concentration was determined using a haemocytometer and adjusted to 2 × 10^7^ spore mL^−1^.

The mycorrhizal formulation Miratext-02, provided by Mirat Fertilizantes (Salamanca, Spain), was used as the AMF inoculum, which contained at least 1 × 10^6^ spore kg^−1^ in an inert substrate and included five different AMF species: [*Glomus microagregatum*, *Funneliformis mosseae* (formerly *G*. *mosseae*), *Claroideoglomus claroideum* (formerly *G*. *claroideum*), *Rhizophagus irregularis* (formerly *G*. *intraradices*) and *R*. *fasciculatus* (formerly *G*. *fasciculatum*)].

### Fungal inoculation and plant growth conditions

Seeds were plated on Murashige and Skoog (MS) (Duchefa, Haarlem, The Netherlands) solid medium (agar 1%) with sucrose (1%) and plates mantained in a growth chamber at 22 °C, 40% relative humidity (RH) and a 16 h light/8 h dark photoperiod at 80–100 μE m^−2^ s^−1^, for 7 (rapeseed), 10 (arabidopsis) and 16 (tomato) days. Seedlings were individually transferred to 0.2 (for arabidopsis) and 5 (for rapeseed and tomato) L-pots, containing a mixture of peat/vermiculite (3:1), maintained under greenhouse conditions as previously described^57^ and watered as needed. For each type of plant, the four considered treatments were: untreated (control) and plants treated with AMF, T34, and AMF plus T34. The inoculation of AMF was done by burying 1 g of Miratext-02 (1000 spore g^−1^) per pot at 5 cm below the substrate surface just before transplanting the seedlings. Plants treated with *T*. *harzianum* T34 were root inoculated with 1 mL of a conidial suspension containing 2 × 10^7^ spore mL^−1^ one week after the seedlings have been transplanted. Twenty plants were used for each type of plant and treatment, and each assay was repeated three times.

The siliques from 15 arabidopsis and rapeseed plants per condition were collected at the end of the life cycle and counted (11 weeks for arabidopsis and 19 weeks for rapeseed). The analyses of fungal-root colonization and the expression of defense genes were performed with roots of a total of 60 plants of arabidopsis, rapeseed and tomato. For each type of plant, roots from five plants per each treatment were pooled and root pools from three independent assays were considered. Roots were collected one week after T34 inoculation and during the formation of the floral primordia in 5- (arabidopsis), 10- (rapeseed) and 7- (tomato) week-old plants, except in the case of the arabidopsis mutants since *sid2* mutant was unable to reach reproductive growth in the presence of T34^32^. In this last case, roots were collected 10 days after fungal inoculation when the plants were 4 weeks old. All root material was washed with water until there was no remaining substrate, immediately frozen with liquid nitrogen and pulverized with a mortar.

### Quantification of fungal root colonization

The quantification of T34 and AMF DNA in the roots of rapeseed, arabidopsis and tomato plants was performed by qPCR as previously described^62,63^, with some modifications. DNA was extracted from roots of the untreated (control) and AMF, T34, and AMF plus T34 inoculated plants. A mix was prepared in a 10-μL volume using 5 μL of Brilliant SYBR Green QPCR Master Mix (Roche, Penzberg, Germany), 10 ng of DNA, the forward and reverse primers at a final concentration of 100 nM, and nuclease-free PCR-grade water to adjust the final volume. The 18S rRNA gene of AMF^64^ and the *Actin* genes of *Trichoderma*, arabidopsis, rapeseed and tomato were used; their corresponding primer pairs are indicated in Table 3. Amplifications were performed in an ABI PRISM 7000 Sequence Detection System (Applied Biosystems, Foster City, USA) programmed for 40 cycles under the following conditions: denaturation, 95 °C for 15 s; annealing, 60 °C for 1 min; extension, 72 °C for 1 min. Each PCR was performed in triplicate by using the DNA extracted from 3 root pools of 5 plants each one for each treatment and plant type. Cycle threshold values served to calculate the amount of fungal DNA using standard curves. Values of *Trichoderma* or AMF DNA were referred to the amount of arabidopsis, rapeseed or tomato DNA in every corresponding sample.

**Table 3:** Primers used in this work.

Code	Sequence (5′-3′)	Use	References	R^2^	Slope	Efficiency (%)
Act-T-F	ATGGTATGGGTCAGAAGGA	Endogenous *Trichoderma* gene	^63^	0.993	3.346	99.01
Act-T-R	ATGTCAACACGAGCAATGG					
AML1 (F)	CTTTCGATGGTAGGATAGAGG	18S rRNA to AMFs quantification	^64^	0.988	3.279	101.80
AML2 (R)	ACAACTTTAATATACGCTATTGGA					
Act-Bn-F	CCCTGGAATTGCTGACCGTA	Endogenous rapeseed gene	^67^	0.990	3.285	101.54
Act-Bn-R	TGGAAAGTGCTGAGGGATGC					
Act-At-F	CTCCCGCTATGTATGTCGCC	Endogenous arabidopsis gene	^32^	0.996	3.306	100.67
Act-At-R	TTGGCACAGTGTGAGACACAC					
ICS1-At-F	GATCTAGCTAACGAGAACGG	Synthesis gene of SA in arabidopsis	^32^	0.989	3.283	101.64
ICS1-At-R	CATTAAACTCAACCTGAGGGAC					
PR-1-At-F	GGCTAACTACAACTACGCTG	Response gene to SA in arabidopsis	^32^	0.992	3.292	101.24
PR-1-At-R	GGCTTCTCGTTCACATAATTC					
CAL5-At-F	CTTTGCTGGTTTCAACTCAACTC	Response gene to SA in arabidopsis	^32^	0.991	3.291	101.30
CAL5-At-R	AATGTTTGCTCTCCGTTTCC					
LOX1-At-F	GTAAGCTCTGATGTTACTGATTC	Synthesis gene of JA in arabidopsis	^32^	0.988	3.279	101.83
LOX1-At-R	CTGCGGTTAACGACGTGATTG					
VSP2-At-F	GTTAGGGACCGGAGCATCAA	Response gene to JA in arabidopsis	^68^	0.986	3.274	102.02
VSP2-At-R	TCAATCCCGAGCTCTATGATGTT					
PDF1.5-At-F	GTTGCTCTTGTTCTCTTTGCTGA	Response gene to JA in arabidopsis	^69^	0.997	3.309	100.53
PDF1.5-At-R	CCATGTCTCACTTTCCCTTTTGC					
Act-Sl-R	CACCACTGCTGAACGGGAA	Endogenous tomato gene	^57^	0.987	3.278	101.87
Act-Sl-R	GGAGCTGCTCCTGGCAGTTT					
ICS1-Sl-F	GTTCCTCTCCAAGAATGTCC	Synthesis gene of SA in tomato	^57^	0.984	3.291	102.41
ICS1-Sl-R	TCCTTCAAGCTCATCAAACT					
PR-1-Sl-F	CCTCAAGATTATCTTAACGCTC	Response gene to SA in tomato	^57^	0.983	3.263	102.54
PR-1-Sl-R	TACCATTGCTTCTCATCAACC					
LOX1-Sl-F	GCCTCTCTTCTTGATGGAG	Synthesis gene of JA in tomato	^57^	0.995	3.268	100.75
LOX1-Sl-R	GTAGTGAGCCACTTCTCCAA					
EIN2-Sl-F	GTTGCTAAGTGATGCTGTA	Response gene to JA/ET in tomato	^57, 70, 71^	0.997	3.306	100.48
EIN2-Sl-R	CGCTCAAGCATGCTGGGCC					

The primers used for the quantification of the DNA of each species are sufficiently specific, without showing any of them, cross reaction with any of the other fungal and/or plant species used.

### Gene expression studies

Roots of three groups of 5 arabidopsis or tomato plants per assayed condition were separately collected and used for RNA extraction using the TRI reagent (Ambion, Austin, USA), following the manufacturer’s instructions. The cDNA synthesis was performed as previously described^61^. Gene expression was analyzed by qPCR. PCR mixtures and amplification conditions were as previously described^65^. The primers used are given in Table 3, and the *Actin* gene was used as the arabidopsis and tomato endogenous control. Data are expressed using 2^−ΔΔCT^ method^66^.

### Statistical analysis

The statistical analysis of the data was carried out with the Statistix 8.0 software. When a comparison of means was performed against a control, the Student’s t-test was employed at *P* < 0.05; significant differences are denoted using an asterisk. Combined effects of strain T34 and AMF were analyzed by two-way ANOVA followed by Sidak’s multiple comparison test, indicating significant differences as follows: *P < 0.05; **P < 0.01; ***P < 0.001; ****P < 0.0001.
